# Experimental Model Systems Used in the Preclinical Development of Nucleic Acid Therapeutics

**DOI:** 10.1089/nat.2023.0001

**Published:** 2023-08-09

**Authors:** Haiyan Zhou, Virginia Arechavala-Gomeza, Alejandro Garanto

**Affiliations:** ^1^ Genetics and Genomic Medicine Research and Teaching Department, Great Ormond Street Institute of Child Health, University College London, London, United Kingdom.; ^2^ NIHR Great Ormond Street Hospital Biomedical Research Center, London, United Kingdom.; ^3^ Nucleic Acid Therapeutics for Rare Disorders (NAT-RD), Biocruces Bizkaia Health Research Institute, Barakaldo, Spain.; ^4^ Ikerbasque, Basque Foundation for Science, Bilbao, Spain.; ^5^ Department of Pediatrics, Amalia Children's Hospital, Radboud Institute for Molecular Life Sciences, Radboud University Medical Center, Nijmegen, The Netherlands.; ^6^ Department of Human Genetics, Radboud Institute for Molecular Life Sciences, Radboud University Medical Center, Nijmegen, The Netherlands.

**Keywords:** nucleic acid therapeutics, model systems, cellular models, animal models, antisense technology, efficacy

## Abstract

Preclinical evaluation of nucleic acid therapeutics (NATs) in relevant experimental model systems is essential for NAT drug development. As part of COST Action “DARTER” (Delivery of Antisense RNA ThERapeutics), a network of researchers in the field of RNA therapeutics, we have conducted a survey on the experimental model systems routinely used by our members in preclinical NAT development. The questionnaire focused on both cellular and animal models. Our survey results suggest that skin fibroblast cultures derived from patients is the most commonly used cellular model, while induced pluripotent stem cell-derived models are also highly reported, highlighting the increasing potential of this technology. Splice-switching antisense oligonucleotide is the most frequently investigated RNA molecule, followed by small interfering RNA. Animal models are less prevalent but also widely used among groups in the network, with transgenic mouse models ranking the top. Concerning the research fields represented in our survey, the mostly studied disease area is neuromuscular disorders, followed by neurometabolic diseases and cancers. Brain, skeletal muscle, heart, and liver are the top four tissues of interest reported. We expect that this snapshot of the current preclinical models will facilitate decision making and the share of resources between academics and industry worldwide to facilitate the development of NATs.

## Introduction

Nucleic acid therapeutics (NATs) are one of the fastest growing types of drugs. They treat diseases in a target-specific manner and offer great therapeutic potential for a wide range of disorders, applicable not only to common genetic disorders but also to rare diseases and personalized medicine. Novel NAT strategies using various nucleic acid technologies have been successfully developed, with approvals from the United States of America Food and Drug Administration (FDA) or the European Medicines Agency (EMA) for neuromuscular, neurodegenerative, and metabolic disorders, among others [1,2].

Before a successful clinical translation, preclinical evaluation of NATs in suitable experimental systems is essential. Relevant model systems, including cellular and animal models, are used to evaluate their effectiveness on regulating target gene expression [3], downstream functional readouts [4], the compounds' uptake and biodistribution [5], and to perform potential toxicology studies [6].

**FIG. 1.**
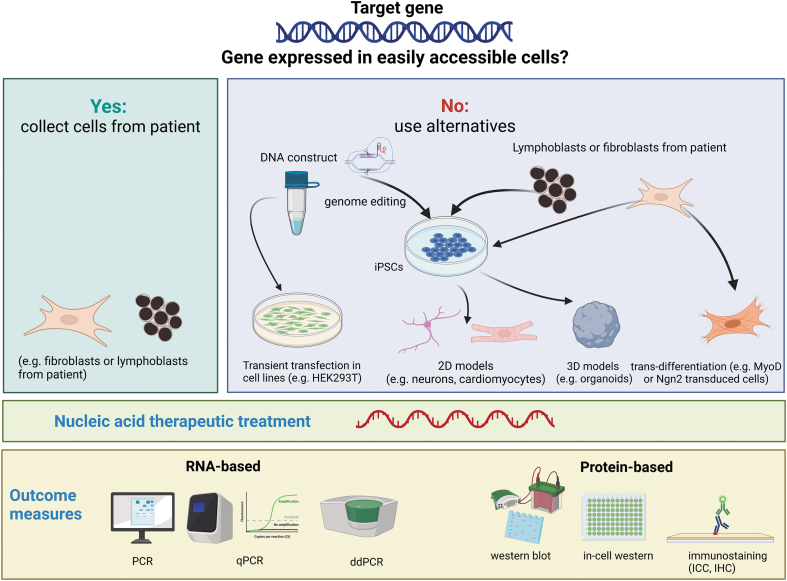
Schematic representation on the selection of the best cellular model when applying NATs. Figure created with Biorender.com. NATs, nucleic acid therapeutics.

Cell-based assays are an essential element of NAT drug discovery. Patient-derived cellular cultures are particularly useful to model human diseases, especially for the mutation-specific NAT approaches [4,7–10]. While relevant disease phenotypes in cellular models pave the way toward high-throughput screening of NAT drugs, maintenance and expansion of human primary cells for large-scale screening remain challenging. Another challenge of using human-derived cells for NAT drug evaluation is the limited cell types available due to the difficulties in tissue accessibility to certain organs. This obstacle can now be overcome by the use of induced pluripotent stem cell (iPSC) technology, which allows subsequent differentiation into diverse cell types [11,12]. This technology has provided the feasibility of allowing NAT drug evaluation in broad type of cells (Fig. 1).

For complex tissues, organoids cultured in 3D may approximate to the target tissues more accurately than the 2D cellular system [13]. Some *ex vivo* 3D models closely recapitulate tissue architecture and cellular composition of the target organs. iPSC-derived organoids can contain cell types derived from all three germ layers [14]. Patient-derived organoids have been used in antisense oligonucleotide (AON)-mediated gene knockdown assays in tumors [15,16] and neurological conditions [17,18]. This “disease-in-a-dish” model presents the potential to predict patient response, hence holding great promise for personalized medicine [13,19–24]. Comprehensive NAT drug evaluation *in vitro* in cellular models enables reliable preliminary screening of potential NAT drugs thus preventing incompetent compounds from entering further validation phase in animal models.

Animal models are also important in NAT drug development. However, as NAT approaches are sequence specific, very often the target sequences in animal models, usually rodents, are different from the human target gene due to sequence variations among species. Therefore, an “animal version” of the NAT drug is usually used for proof-of-concept studies, although this molecule can have different properties compared with the “human version.” Alternatively, a model carrying the human equivalent mutation or a humanized animal model, where the animal gene is completely or partially replaced by the human copy or edited to become more human-like, would be ideal for the *in vivo* NAT validation [25–27]. It is important, however, to ensure that the target gene conducts a similar function in the animal model and that the humanization will not affect its function, especially when aiming to mimic disease and assess functional readouts [28–31].

Most importantly, the use of animal models can provide crucial information on biodistribution, toxicity at specific doses, and the *in vivo* therapeutic efficacy of NAT drugs. This information is pivotal for the translation of NAT drugs to human clinical trials. In some cases, toxicology studies of NAT drugs in nonhuman primates may also be needed before its translation to human trials [32].

With the purpose of bringing together the expertise and sharing knowledge in NAT development across Europe and other associated countries, we created the network “Delivery of Antisense RNA ThERapeutics (DARTER)” (www.antisenserna.eu), which is supported by the European COST Action Program grant nr. CA17103. The network includes researchers with interests in the specific NAT chemistry and modifications, delivery methodology, and a wide range of disorders and target tissues. It is composed of over 350 members representing academia, industry, health systems, and patient advocacy groups. We aim to join forces to further improve NAT as a viable therapeutic option by studying the best ways to deliver these drugs to different target tissues. Model system is one of the key topics that the DARTER network has been focused on. In the last 4 years, this working group has shared among its members their individual experiences in using different models at DARTER seminars and through shared protocols [33].

The DARTER network has recently conducted a survey on the model systems routinely used by members directly involved with preclinical NAT development. A significant proportion of members investigate antisense technology as potential treatments, but also other NAT strategies such mRNA delivery or genome editing, as well as nonviral delivery methods (nanoparticles) are represented. We expect that this report will contribute to clarifying various model systems used in NAT development, especially, but not exclusively, for antisense technology, and promote knowledge and resources sharing not only among members of the DARTER network, but also with academics and industry worldwide to facilitate the development of NATs.

## Methods

The online survey was a Google form distributed in May and June 2021 to the members of DARTER network. The questionnaire consists of questions about the participants (research group and country information), the model systems (three cellular models and three animal models most frequently used in their laboratory), the type of disease(s) investigated, the kind of therapeutic molecules tested, and the read-outs used for evaluation. The blank questionnaire can be found in Supplementary Data S1.

**FIG. 2.**
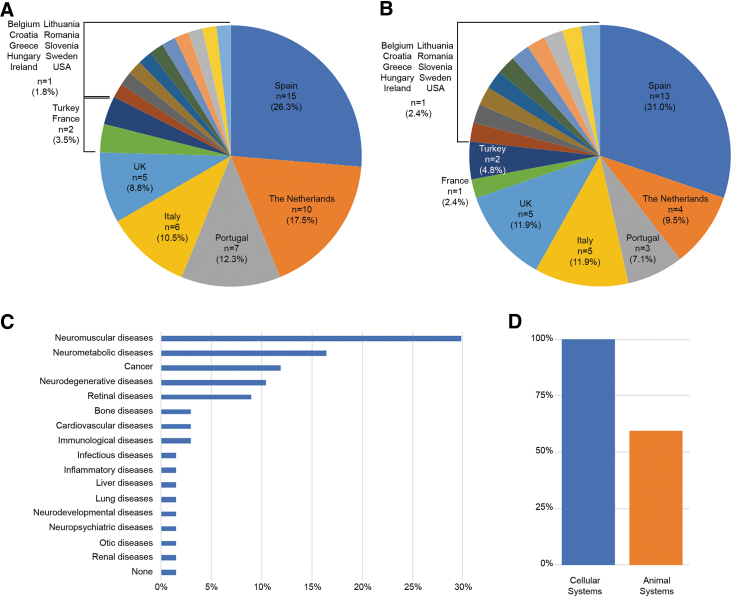
**(A)** Distribution of answers per country (*n* = 57) and **(B)** distribution of research groups per country (*n* = 42). **(C)** Classification of diseases investigated within our DARTER COST Action (total of diseases 67). **(D)** Research groups reporting the use of at least one cellular model (*blue*) or animal model (*orange*). DARTER, Delivery of Antisense RNA ThERapeutics.

In total, we received answers from 57 researchers in 15 European countries, Turkey, and the United States (Fig. 2A). To avoid overrepresentation of any large research groups, we classified the answers based on the group leader of the research team and we obtained answers from 42 independent research groups in 17 countries (Fig. 2B). We then classified the answers by disease groups. Neuromuscular disorders were the most frequently studied diseases within our network (∼30%), followed by neurometabolic diseases (∼16.4%) and cancers (∼12%). In total, 17 groups of diseases were reported (Fig. 2C). It is noted that some research groups investigate multiple diseases. There are two answers on general toxicity upon delivery rather than efficacy in a particular disease, which were not related to any disease and marked as “None” (Fig. 2C). The survey (100%) indicated that all groups use at least one cellular model. In contrast, only 59% of the groups (*n* = 25) use animal models for their studies (Fig. 2D).

## Results

### *In vitro* models

**FIG. 3.**
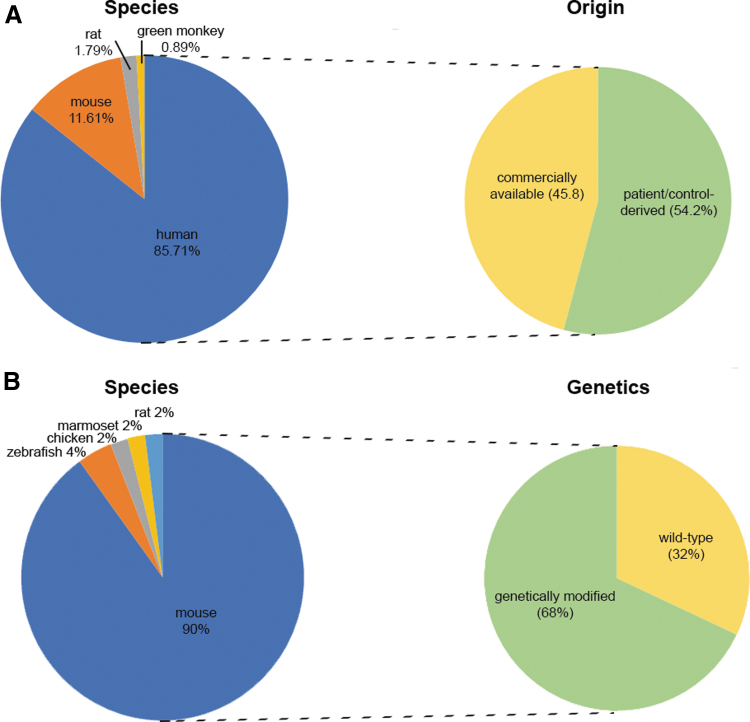
Classification of the species origin of the **(A)** *in vitro* and **(B)** *in vivo* models and whether they are (A) personalized (patient/control-derived) or commercially available cellular models or (B) genetically modified or wild-type animal models.

The main cellular models used by our network members originated from four different species. The most common type of cells used are of human origin (∼85.72%), followed by mouse, rat, and green monkey (∼11.6%, ∼1.8%, and ∼0.89%, respectively) (Fig. 3A). When human cells were reported, ∼54% of the answers mentioned patient/healthy donor-derived cells, while the rest were commercially available cells (∼46%). In this survey, those tumor-derived cell lines, such as HeLa, Neuroblastoma, or WERI-Rb1 have been categorized as commercially available cells (Fig. 3A).

In total, 113 cellular models were reported, which contained 67 unique entries (Supplementary Table S1). Skin-derived fibroblasts were the most frequently used cell type by respondents to our survey (∼15% of the answers), followed by the kidney cell line HEK293T (human embryonic kidney 293T) reported by ∼6.2% of respondents. Interestingly, six of the cultures reported (∼7.1%) in other categories were differentiated from iPSCs. Regardless of the type of cells obtained, iPSC-derived models accounted for ∼11.6% of all lines (including undifferentiated iPSCs as a model itself), highlighting the potential of this technology in the preclinical development of NAT.

Furthermore, 3D cellular models represented only ∼4.4% of the answers, while as unique entries, this percentage was increased up to ∼7.5%. Finally, we classified the unique entries into the tissue of origin (Supplementary Fig. S1A). As expected, this allowed us to reduce the entries to 20. Once all the different cellular models were classified by tissue of origin, muscle cells became the most frequent category with ∼22.4% of the responding laboratories using this model (Supplementary Fig. S1B). This translated into ∼19.5% of the total answers, which is supported by the large number of researchers investigating neuromuscular diseases within our network. Skin models, accounting for 17.7% of the answers, was the second most reported model, however, as a unique entry, skin models dropped to the sixth position representing 5.97% of all unique models (Supplementary Fig. S1). This is partly explained by the fact that fibroblasts were counted as a single model system from skin origin. The third category referred to neuronal model systems with ∼10.6% of the answers and 13.43% of the models.

**FIG. 4.**
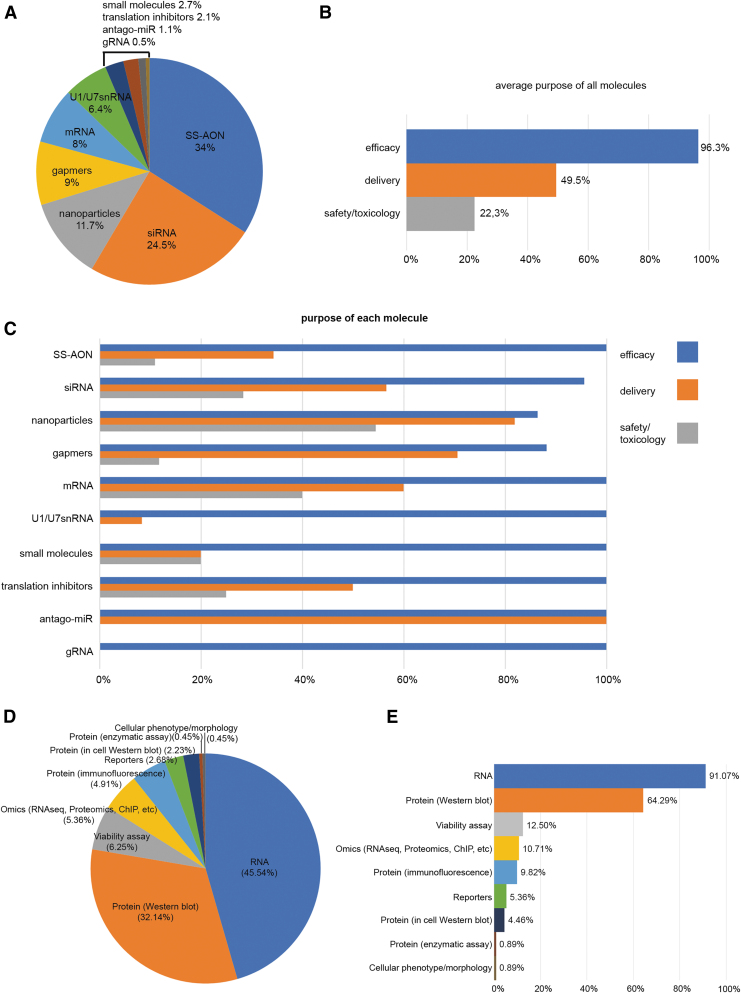
Graphical representation of the percentage of molecules and readouts assessed in cellular models. **(A)** Percentage of the molecules with respect to all the answers. **(B)** The study purpose for all the molecules and **(C)** the specific purposes for efficacy, delivery, and safety/toxicology for every type of therapeutic molecule. **(D)** Percentage of different readouts from the total amount of answers and **(E)** the type of readout performed in each line described in the survey, expressed as percentage (eg, in 91.07% of the reported cell lines, a readout at RNA level is conducted).

Concerning the purpose behind the use of these models and which type of molecules are routinely assessed for NAT development, 10 types of molecules were reported, from which splice-switching AONs (SS-AON) and small interfering RNA (siRNA) together accounted for >50% of the answers (Fig. 4A). Most of these molecules were used to assess efficacy (96.3%), followed by evaluation of delivery (49.5%) or safety/toxicology (22.3%), as shown in Fig. 4B. When the results were segregated by the type of molecule (Fig. 4C), it was apparent that efficacy was evaluated in all of them. As expected, for respondents working with nanoparticles, the delivery assessment was almost equally important to the efficacy evaluation in ∼80% of the answers. At the same time, researchers working with nanoparticles showed a high interest in safety and toxicology (>50%), whereas those studying UsnRNA systems (U1 and U7) were barely interested in delivery and safety/toxicology. The very low number of entries mentioning small molecules, translation inhibitors, antago-miRs, and gRNAs (5, 4, 2, and 1, respectively) precluded the identification of any clear trends related to these molecules.

Finally, we asked our members which readouts were usually used to evaluate efficacy in each of the cell lines they reported. As expected, RNA and protein expression were the two major readouts accounting for ∼85% of all the answers (Fig. 4D). When looking at the readouts for each cell line, in almost all cases the major readout is the response at RNA level, regardless of the cell line studied (Fig. 4E). Again, the second most common readout is protein assessment by western blot or other methods [34]. Overall, these results are in line with the current practices that if no effect at RNA level is observed, the molecule is considered not efficacious and therefore further studies are not pursued. Only if lead molecules are effective at RNA level, further validation will be pursued. This could explain the difference between RNA and protein analyses (91% vs. 64%).

### *In vivo* models

In total, 59% of the groups reported the use of at least one animal model. Remarkably, mouse models were the most frequently employed model system within DARTER. The other four models mentioned are zebrafish (4%), chicken embryos (2%), marmoset (2%), and rat (2%). Around 68% of the listed animal models were genetically modified, while ∼32% were wild type (Fig. 3B).

**FIG. 5.**
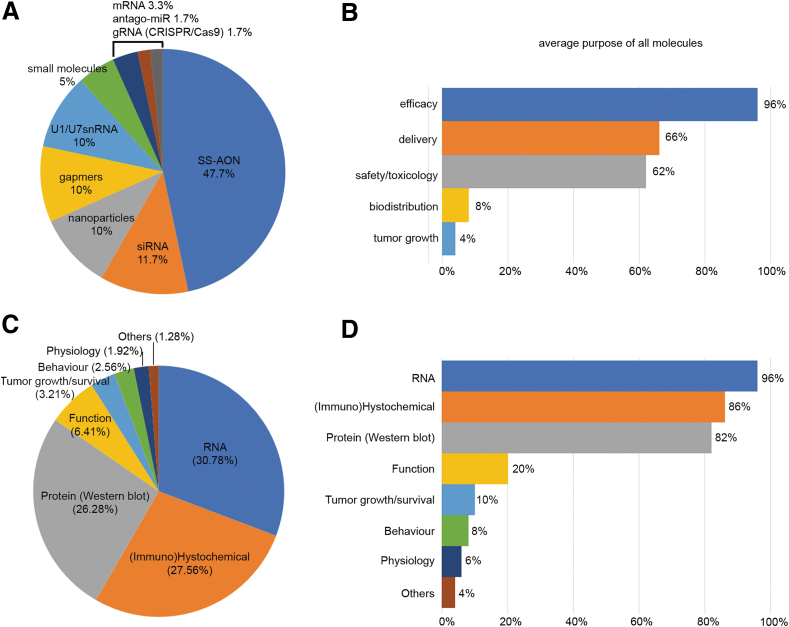
Graphical representation of the percentage of molecules and readouts assessed in animal models. **(A)** Percentage of the molecules with respect to all the answers. **(B)** The study purpose in each animal model expressed in percentage (eg, 96% of the models were used for efficacy of a therapeutic molecule). **(C)** Percentage of all readouts with respect to the total amount of answers. **(D)** The type of readout performed in each animal model described in the survey, expressed as percentage.

Similar to the *in vitro* models, SS-AONs were the most frequently evaluated molecules (∼46.7%) *in vivo* (Fig. 5A). They were followed by siRNA (∼11.7%), gapmers (∼10%), nanoparticles (∼10%) and U1/U7snRNA systems (∼10%). Other reported molecules included mRNA, small molecules, antago-miRs or gRNAs (CRISPR/Cas9 system). In 96% of the answers, the molecules were assessed in animal models to evaluate the efficacy (Fig. 5B). Delivery and safety/toxicology were also highly indicated with 66% and 62% of the models being used for these purposes. Biodistribution only represented 8% of the answers.

Thirteen delivery routes were reported in the *in vivo* model systems. Intravenous injection was the most preferred administration route with around 32% of respondents, followed by intracerebroventricular and subcutaneous injections (∼18% and ∼11% of answers, respectively). In general, by classifying the answers into local and systemic delivery, the percentages were similar at ∼45% and ∼55%, respectively. When questioned on which tissues/organs were of interest, brain (∼21.7%), muscle (∼19.1%), heart (∼16.5%), and liver (∼14.8%) were the top four answers (Supplementary Fig. S2). This is in line with the distribution of the diseases studied in our network. Compared with cellular models, no specific strain or model was recurrently used over others. However, in general, the majority of the mouse models reported were used to study neuromuscular diseases, in particular associated with Duchenne and Becker muscular dystrophy.

Regarding the type of readouts used in animal models, and similar to the *in vitro* data, RNA analyses were still the major measurement accounting for ∼30.8% of the answers and applied to ∼96% of the models described. This is followed by (immune)histochemical analysis and protein analysis by western blotting, reported in ∼27.6% and ∼26.3% of the answers and applied to ∼86% and ∼82% of the models, respectively. Other readouts used were functional assessment, tumor growth/survival, and behavioral and physiological analyses (Fig. 5C, D).

## Discussion

The procedure for preclinical development of a therapeutic molecule usually involves an initial step with a series of assays performed in cellular model systems, which also applies to NATs. Once the nucleic acid sequences are designed using *in silico* predictions [35–37], they are subsequently assessed on efficacy in cellular models [33]. Thereby, having a suitable cellular model system is crucial not only at initial development stages, but also for lead candidate selection and optimization. The results of the survey are in line with this purpose. As discussed in our previous review, delivery of NATs to target cells and tissues is an important issue in NAT development [1], and this survey also included reports of nanoparticle evaluations. Furthermore, it is a common practice to exclude molecules that are highly toxic or low efficient in cell culture from further evaluations. In that sense, only the safest and most efficacious molecules will be taken forward to thorough safety and toxicology studies performed in animal models.

In our survey, mice appeared to be the first choice as an *in vivo* model, probably due to the fact that mice are easy to manipulate genetically, maintain and breed, and while sharing more genetic homologies with human than other commonly used experimental animal models such as fruit fly and zebrafish. It is also necessary to note that many mouse models have already been generated and characterized in the past, and the delivery routes and readouts are also established.

NATs are often directed toward a specific sequence either in patient's DNA, pre-mRNA, or mRNA. Thus, the selection of the *in vivo* model system to assess efficacy should be determined by the target expression. The most frequently used cell lines reported in the survey are from human origin. Although the commercially available human cell lines offer easy accessibility to models highly accepted in the scientific field, these cell lines lack the patient-specific characteristics, such as the pathogenic variant or the molecular defect. While patient-derived material would be a better model, it is not always easy to obtain. Current implementation of advanced genetic diagnostic techniques using patients' blood (considered a noninvasive approach), has made the requirement of tissue biopsies as diagnostic material redundant and the availability of spare tissue has decreased enormously.

Thus, when choosing the model system to study NATs in a disease, it is important to take into consideration the following criteria: (1) the gene/target of interest is expressed in the particular cell line, (2) the nucleic acid molecule is directed to the same gene/target of the species of origin of the line, (3) the cell line can be cultured, (4) delivery of NAT molecule is feasible, and (5) if the mutation-specific effect is recapitulated in this model.

Two particular cell types were recurrently reported in our survey: HEK293T and skin fibroblasts. As a conventional cell line from human embryonic kidney, HEK293T cells offer the possibility to perform experiments requiring large number of cells in a relatively short period of time. When the gene of interest is not present, vectors containing the target gene can be transfected into HEK293T cells as an experimental cellular model for NAT studies, for example on splice switching or gene silencing [3,38,39]. This system however, often relies on the overexpression of part of the gene and lacks the entire gene context (such as introns, splicing enhancers or inhibitors) and, therefore, may lead to different results between the artificial and real situations or even between different cell types [11,40,41].

The DARTER network continues the work of a previous European COST action called “Exon skipping” (Number BM1207), which included many researchers on neuromuscular disorders, as many first-in-man studies had been conducted in this field [42]. This may explain the bias in our current survey toward neuromuscular disorders and skeletal muscle. Among the muscle models reported, myoblasts and myotubes were the cell types most frequently used. In the case of genes only expressed in differentiated muscle cells (myotubes), researchers need to differentiate myoblasts to myotubes. It is hence necessary to report what protocols were used in culture and differentiation, and to compare results between different laboratories. When muscle culture is not available, fibroblasts are sometimes used by researchers as an alternative. Similar situations are also experienced when other organs are studied. This makes fibroblast lines a convenient model widely used among our members, independent of disease pathology.

Dermal fibroblasts generated from skin biopsies used to be part of the routinely performed standard procedure of many biobanks. Fibroblasts allow studies in the precise genetic background of the patient where the pathogenic variant is present. However, as skin-derived cells, dermal fibroblasts do not express genes that are tissue specific in other organs. To circumvent this issue, cell transformation may be performed, for example transdifferentiate dermal fibroblasts to muscle or neuronal cells using MyoD or NGN2 overexpression [43], or reprogrammed into iPSCs using the four Yamanaka vectors [11,12]. iPSC technology has revolutionized the field and nowadays we can differentiate those cells into almost any cell type of the human body. Although iPSC technology is costly and time-consuming, several groups within our network have shown the potential of these models in assessing NAT treatment, in particular for eye and brain diseases [10,19,23,40,44,45].

In addition, gene editing techniques such as CRISPR/Cas9 can insert specific mutations in iPSCs and primary control lines [46], allowing the study of the direct effect of the mutation and obtaining a line that would mimic the condition of patient [47,48]. This approach has provided a powerful tool for generating specific mutant cell lines for NAT development [49]. Overall, primary skin fibroblasts cultured from patients with rare diseases are valuable bioresources for NAT development as highlighted in our survey. Therefore, strategies to connect biobanks and researchers are important to continue investigating treatments for rare diseases.

In our survey, the majority of cultures used were in 2D systems, although the target organs and tissues are organized in a more complex 3D structure. iPSC technology enables the generation of 3D models in the form of organoids, with a structure more closely resembling the tissue of interest than the 2D models. However, this is usually a laborious and lengthy procedure that, for example, takes from >80 days to form brain organoids and 200 days for retinal organoids.

Organ-on-chip is a technology aiming to combine different tissues or cell types to study disease and test therapeutics in a complex environment similar to *in vivo*. Examples include the blood–brain–retinal barriers on chip connected to microfluidic chambers that even allow multiplexing [50,51]. The development of these systems may allow the identification of chemical compounds for systemic delivery able to cross the blood barriers of the brain or the retina, accelerating the development and reducing the number of experimental animals used for this initial identification [13,20,24]. The 3D disease modeling system has potential not only in disease mechanism study but also for *in vitro* drug screening, hence accelerate novel NAT development.

Despite the options of numerous cellular models aforementioned, in many conditions, animal models are still required for pharmacological testing of NAT. The use of the animal models, mainly mice and rats, provides important information on pharmacokinetics, biodistribution, safety and toxicity at specific doses, and therapeutic efficacy. This information is important for the translation of the molecules to clinical trials.

Since NAT molecules are sequence specific, very often the target sequence in the rodent models is different from the human target gene, due to sequence variation between species. To overcome this hurdle, a humanized rodent model where the target gene is replaced or partially replaced by the human counterpart could be used for the *in vivo* validation of the human NAT sequence. Deep phenotyping is required to assess whether the humanization recapitulates the disease phenotype or maintains the function of the gene [26,52,53]. Second to mouse models, zebrafish is also reported in our survey, likely due to the convenient genetic modification, well characterized development, high capacity, and rapid turnaround as a conventional experimental animal model [54].

## Conclusions

In conclusion, both cellular and animal models are required for NAT development. While the conventional cellular models are still widely used by researchers, newly developed model systems, such as iPSC-differentiated cells, CRISPR/Cas9 gene editing-induced disease cellular model and organ-in-chip 3D model are expanding rapidly in this field. These advanced or more complex *in vitro* model systems may not only overcome the shortage of patient-derived primary cellular models, but also function as a surrogate model to reduce the number of animals used in the subsequent *in vivo* evaluations.

The selection of a suitable model system should be based on the research question that needs to be answered, and how reliable the model in recapitulating the human condition. Only taking together the results of orthogonal methods and models will provide reliable information about the NAT molecule. Several guidelines have been published on experimental design of NAT studies [7,55]. It is hence important to have standardized protocols for evaluation of NATs in different model systems [56,57]. This requires international efforts to establish guidelines to facilitate the preclinical development of NATs.

The DARTER network is working together with groups worldwide to develop guidelines on how to develop NATs. One of the main efforts is the establishment of standardized protocols and uniformed evaluating systems. However, we acknowledge that with different NAT modalities and variety of target organs, each model needs to be selected specifically based on the research question that needs to be addressed.

## Supplementary Material

Supplemental data

Supplemental data

Supplemental data

Supplemental data
