# Supplementary material for: Experimental Model Systems Used in the Preclinical Development of Nucleic Acid Therapeutics
Source: Nucleic Acid Ther. 2023 Aug 9;33(4):238–47. doi: 10.1089/nat.2023.0001 (PMC10457615; doi:10.1089/nat.2023.0001)
Supplement: Supplemental data [file Suppl_DataS1.pdf]

Supplementary File 1

Name and Surname(s) *

Your answer

Contact email *

Your answer

Country (you are based in) *

Your answer

PI of the research group *

Your answer

Disease of interest *

Your answer

Cellular models
Please describe the cellular models you use the most in your research to a maximum of 3 options. Please use the different boxes for each cell line. It is not needed to provide three cellular models, but some labs may use 2 or more complementary model systems. Please only describe the models you would consider yourself an expert.

Cellular model #1

Cell line #1

Your answer

Purpose

- [ ] Efficacy
- [ ] Safety/Toxicology
- [ ] Delivery
- [ ] Other:

Type of molecule assessed

- [ ] splice switching oligonucleotides
- [ ] gapmers
- [ ] siRNA
- [ ] mRNA
- [ ] nanoparticles
- [ ] Other:

Type of analysis/readout

- [ ] RNA
- [ ] Protein (Western Blot)
- [ ] Other:

Cellular model #2
Optional

Cell line #2

Your answer

Purpose

- [ ] Efficacy
- [ ] Safety/Toxicology
- [ ] Delivery
- [ ] Other:

Type of molecule assessed

- [ ] splice switching oligonucleotides
- [ ] gapmers
- [ ] siRNA
- [ ] mRNA
- [ ] nanoparticles
- [ ] Other:

Type of analysis/readout

- [ ] RNA
- [ ] Protein (Western Blot)
- [ ] Other:

Cellular model #3
Optional

Cell line #3

Your answer

Purpose

- [ ] Efficacy
- [ ] Safety/Toxicology
- [ ] Delivery
- [ ] Other:

Type of molecule assessed

- [ ] splice switching oligonucleotides
- [ ] gapmers
- [ ] siRNA
- [ ] mRNA
- [ ] nanoparticles
- [ ] Other:

Type of analysis/readout

- [ ] RNA
- [ ] Protein (Western Blot)
- [ ] Other:

Animal models
Please describe the animal models you use the most in your research to a maximum of 3 options. Please use the different boxes for each model. It is not needed to provide three models. Please only describe the models you would consider yourself an expert.

Animal model #1

Model (provide in this order: species, genetic modification (if known), website (if commercially available) or PMID for the publication describing it)

Your answer

Organ(s) of interest

- [ ] Brain
- [ ] Eye
- [ ] Heart
- [ ] Kidney
- [ ] Liver
- [ ] Lung
- [ ] Muscle
- [ ] Skin
- [ ] Other:

Type of delivery (write full name, not abbreviations)

Your answer

Purpose

- [ ] Efficacy
- [ ] Safety/Toxicology
- [ ] Delivery
- [ ] Other:

Type of molecule assessed

- [ ] splice switching oligonucleotides
- [ ] gapmers
- [ ] siRNA
- [ ] mRNA
- [ ] nanoparticles
- [ ] Other:

Type of analysis/readout

- [ ] RNA
- [ ] Protein (Western blot)
- [ ] (Immuno)Hystochemical
- [ ] Other:

Animal model #2
Optional

Model (provide in this order: species, genetic modification (if known), website (if commercially available) or PMID for the publication describing it)

Your answer

Organ(s) of interest

- [ ] Brain
- [ ] Eye
- [ ] Heart
- [ ] Kidney
- [ ] Liver
- [ ] Lung
- [ ] Muscle
- [ ] Skin
- [ ] Other:

Type of delivery (write full name, not abbreviations)

Your answer

Purpose

- [ ] Efficacy
- [ ] Safety/Toxicology
- [ ] Delivery
- [ ] Other:

Type of molecule assessed

- [ ] splice switching oligonucleotides
- [ ] gapmers
- [ ] siRNA
- [ ] mRNA
- [ ] nanoparticles
- [ ] Other:

Type of analysis/readout

- [ ] RNA
- [ ] Protein (Western blot)
- [ ] (Immuno)Hystochemical
- [ ] Other:

Animal model #3
Optional

Model (provide in this order: species, genetic modification (if known), website (if commercially available) or PMID for the publication describing it)

Your answer

Organ(s) of interest

- [ ] Brain
- [ ] Eye
- [ ] Heart
- [ ] Kidney
- [ ] Liver
- [ ] Lung
- [ ] Muscle
- [ ] Skin
- [ ] Other:

Type of delivery (write full name, not abbreviations)

Your answer

Purpose

- [ ] Efficacy
- [ ] Safety/Toxicology
- [ ] Delivery
- [ ] Other:

Type of molecule assessed

- [ ] splice switching oligonucleotides
- [ ] gapmers
- [ ] siRNA
- [ ] mRNA
- [ ] nanoparticles
- [ ] Other:

Type of analysis/readout

- [ ] RNA
- [ ] Protein (Western blot)
- [ ] (Immuno)Hystochemical
- [ ] Other:
