# Supplementary material for: Experimental Model Systems Used in the Preclinical Development of Nucleic Acid Therapeutics
Source: Nucleic Acid Ther. 2023 Aug 9;33(4):238–47. doi: 10.1089/nat.2023.0001 (PMC10457615; doi:10.1089/nat.2023.0001)
Supplement: Supplemental data [file Suppl_TableS1.pdf]

Table S1 – List of unique cell line entries after correcting per research group

Unique entry	Percentage answers
4T1	0.9%
A2780	0.9%
AB1190	0.9%
AB943	0.9%
C2C12	1.8%
COS7	0.9%
Dental Pulp Stem Cells	1.8%
Epithelial cells	0.9%
Fibroblasts	15.0%
Fibromyod	0.9%
H2K	2.7%
HCC2998 colorectal cancer cells	0.9%
HCT-15	0.9%
HEI-OC1	0.9%
HEK293T	6.2%
HeLa	2.7%
Hep3B	1.8%
Hepatocytes	0.9%
Hepatoma cell line	0.9%
HepG2	2.7%
HTB38 colorectal cancer cells	0.9%
hTERT-RPE1	0.9%
HuH7	0.9%
Human neuroblastoma cells (SH-SY5Y)	2.7%
Human pancreatic beta cell line	0.9%
iMCD3	0.9%
Immortalized human myoblasts	3.5%
Immortalized mouse muscle cells	0.9%
Intervertebral disc progenitor	0.9%
Intestinal organoids	0.9%
Intestinal cells	0.9%
iPSC	4.4%
iPSC-derived brain organoids	0.9%
iPSC-derived human islets	0.9%
iPSC-derived neurons	0.9%
iPSC-derived photoreceptor precursor cells (PPC)	1.8%
iPSC-derived retinal organoids	1.8%
iPSC-derived RPE	0.9%
JHH6	0.9%
Jurkat E6.1	0.9%
KM (human myocyte)	1.8%
Km155	0.9%

L6 skeletal muscle cells	0.9%
Liver organoids	0.9%
M14K, malignant pleural mesothelioma	0.9%
MDA-MB-231	1.8%
Mesenchymal stromal cell (MSC)	0.9%
Mouse neuroblastoma (N1E-115-1)	0.9%
Myocyte	0.9%
NCI-H520, squamous cell carcinoma (lung)	0.9%
Neural Progenitor Cells	0.9%
Neuro2A	0.9%
Neuroblastoma	1.8%
Osteoblast	0.9%
Osteoclast	0.9%
PC-12 (mouse pheochromocytoma)	0.9%
PLC	0.9%
Primary cardiomyocyte	0.9%
Primary human islets	0.9%
Primary human myoblasts	0.9%
primary human skeletal muscle cells	0.9%
Primary muscle	0.9%
Primary rat neuron	0.9%
RAW264 macrophages	0.9%
RKO	0.9%
Satellite cells	0.9%
U251-MG - human glioblastoma	0.9%
WERI-Rb-1	0.9%
