# Supplementary material for: Experimental Model Systems Used in the Preclinical Development of Nucleic Acid Therapeutics
Source: Nucleic Acid Ther. 2023 Aug 9;33(4):238–47. doi: 10.1089/nat.2023.0001 (PMC10457615; doi:10.1089/nat.2023.0001)
Supplement: Supplemental data [file Suppl_FigureS1.pdf]

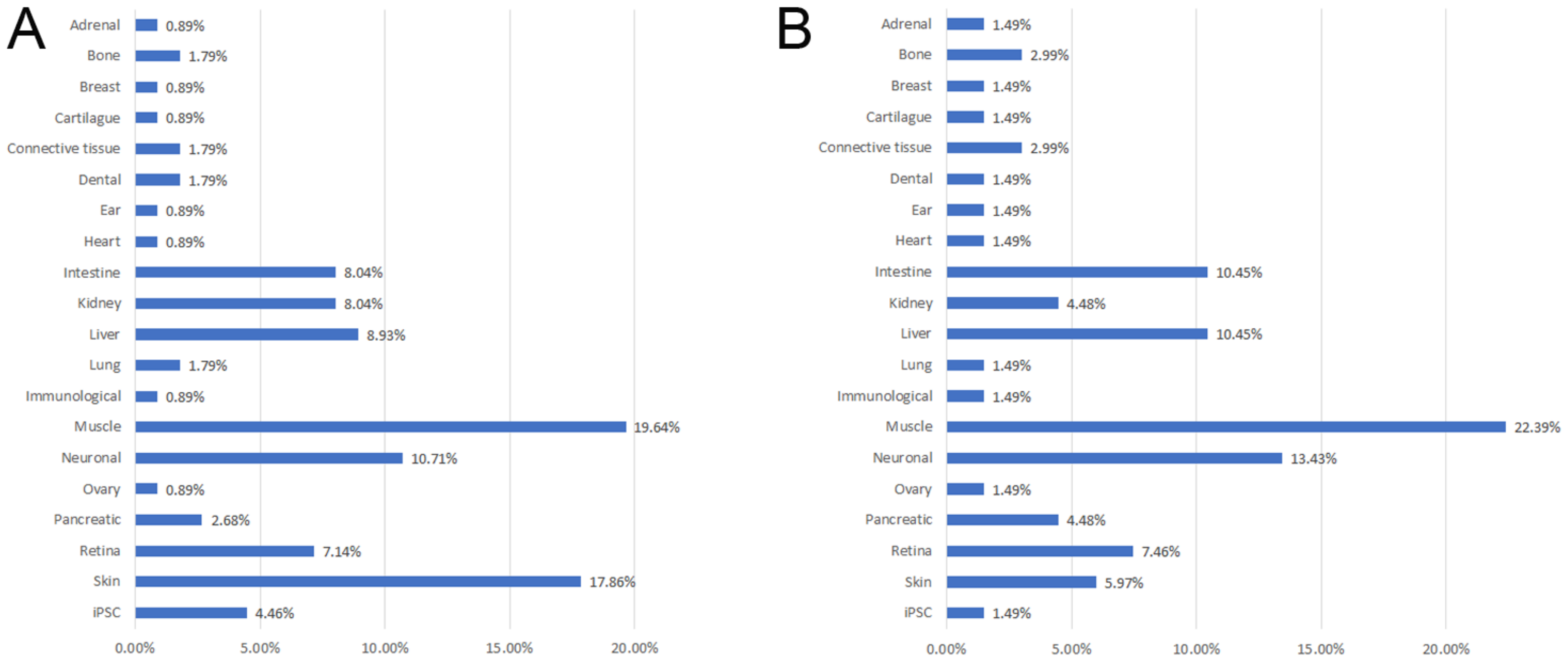

Supplementary Figure S1 – Distribution per tissue of the different cell lines based on the total number of answers (A) or per unique entries (B).
