# Supplementary figures and images for: Experimental Model Systems Used in the Preclinical Development of Nucleic Acid Therapeutics
Source: Nucleic Acid Ther. 2023 Aug 9;33(4):238–47. doi: 10.1089/nat.2023.0001 (PMC10457615; doi:10.1089/nat.2023.0001)

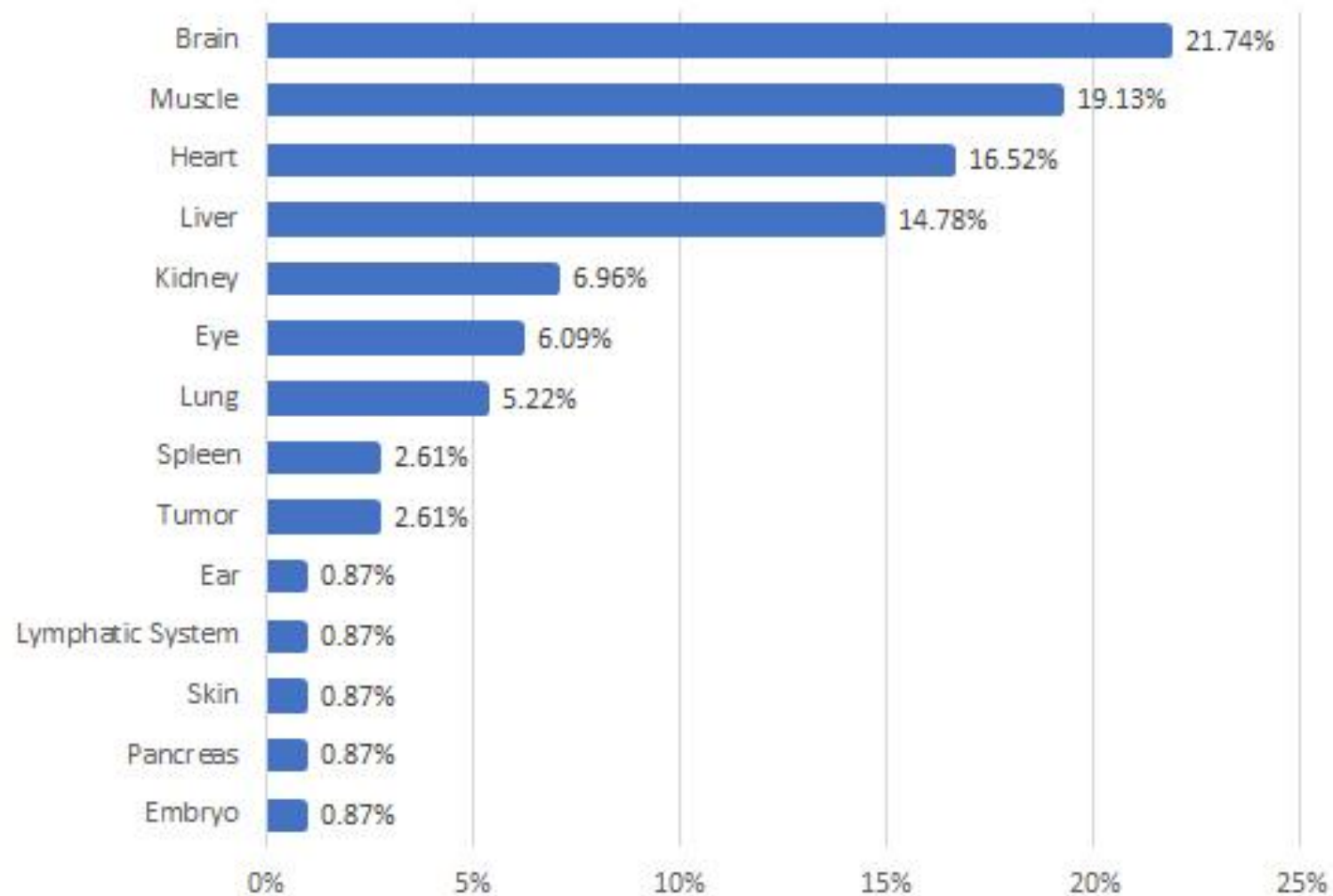

Supplementary Figure S2 – Distribution of the tissues of interest in animal models.

Supplement: Supplemental data [file Suppl_FigureS2.pdf]
